# Trophic Positions of Polyp and Medusa Stages of the Freshwater Jellyfish *Craspedacusta sowerbii* Based on Stable Isotope Analysis

**DOI:** 10.3390/biology12060814

**Published:** 2023-06-03

**Authors:** Sabine Gießler, Tido Strauss, Katrin Schachtl, Thomas Jankowski, Ramona Klotz, Herwig Stibor

**Affiliations:** 1 Faculty of Biology, Aquatic Ecology, Ludwig-Maximilians-Universität München, Grosshaderner Str. 2, 82152 Planegg-Martinsried, Germany; 2 Research Institute for Ecosystem Analysis and Assessment (gaiac), Kackertstrasse 10, 52072 Aachen, Germany; strauss@gaiac-eco.de; 3 Limnological Institute, University of Konstanz, Mainaustraße 252, 78467 Konstanz, Germany; thomas.jankowski@posteo.de

**Keywords:** freshwater jellyfish, *Craspedacusta*, polyp, medusa, *Hydra*, fish, isotopic niche, interspecific competition, benthic and pelagic food web

## Abstract

**Simple Summary:**

Reports of invasive species have increased dramatically in recent decades, raising public awareness. While human activities favor their spread, global warming is believed to promote the establishment of alien species and pose a major threat to native ecosystems. The freshwater jellyfish *Craspedacusta* is an example of a globally successful spread, attracting public attention with its short-lived, eye-catching medusa blooms. Two life stages of this jellyfish are predators: the benthic polyps and the planktonic medusae. Both must meet the requirements in their new food webs to become established. We compared their niches with those of presumed native competitors and found a strong overlap for the medusa stage, suggesting high competition with native zooplanktonic predators and young fish. In contrast, the polyps of invasive *Craspedacusta* and native *Hydra* differed in their niches, possibly favoring a long-term invasion success of *Craspedacusta.*

**Abstract:**

When species spread into new regions, competition with native species and predatory–prey relationships play a major role in whether the new species can successfully establish itself in the recipient food web and become invasive. In aquatic habitats, species with a metagenetic life cycle, such as the freshwater jellyfish *Craspedacusta* with benthic polyps and planktonic medusae, have to meet the requirements of two distinct life stages occurring in two habitats with different food webs. Here, we examined the trophic position of both life stages, known to be predatory, and compared their niches with those of putative native competitors using stable isotope analysis. We found that δ^13^C and δ^15^N signatures of medusae overlapped with those of co-occurring *Chaoborus* larvae and juvenile fish (*Rutilus rutilus*) in a well-studied lake, implying high competition with these native predators. The comparison of δ^15^N signatures of *Hydra* and *Craspedacusta* polyps in four additional lakes revealed their similar trophic position, matching their predatory lifestyle. However, their δ^13^C signatures differed not only across all four of the lakes studied but also within one lake over time, suggesting a preference for pelagic or benthic food sources. We conclude that invasive and native polyps differ in their niches due to different food spectra, which favors the invasion success of *Craspedacusta*.

## 1. Introduction

The freshwater jellyfish *Craspedacusta sowerbii* is a prominent example of a worldwide successful invasion within a century. *C. sowerbii* was first detected in a water-lily tank in the gardens of the Botanical Society in London in 1880 [1] and presumably originates from China [2]. Even though deliberate introductions have not been described, this freshwater invertebrate (phylum Cnidaria, class Hydrozoa, order Limnomedusa) managed to invade all the continents apart from Antarctica [3,4,5]. Not only does its ability to colonize new habitats surpass that of other freshwater medusa species, but it is also one of the most widespread freshwater invaders in the world [6]. *C. sowerbii* is probably even more widespread than currently noted, and many first-time records from countries worldwide since the year 2000 [7,8,9,10,11,12,13,14,15,16,17,18] suggest a continued expansion of its range.

The establishment of invasive species relies on the fulfillment of fundamental niche requirements such as available resources and appropriate environmental conditions [19]. However, invasive species also need a realized niche within the theoretical frame of the fundamental niche to the effect that biotic interactions such as predation or interspecific competition do not prevent the successful establishment of invasive species in otherwise suitable environments [20]. Competition with native species with similar niche requirements could, for example, result in unsuccessful invasion processes [21] because some degree of niche differentiation is necessary to allow for long-term coexistence [22]. Examples of such mechanisms that potentially enable competing species to coexist are dietary segregation associated with differences in feeding behavior [23], resource partitioning based on morphology [24], or spatiotemporal segregation in periods of high resource use [25].

In species with different life history stages, such as *Craspedacusta* with benthic polyps and free-swimming medusae, these mechanisms affect competitiveness during their life cycle. Niche differentiation of at least one of its life stages is a prerequisite for its wide distribution. In addition to the conspicuous pelagic medusa, there are inconspicuous benthic life stages (polyps, frustula, and podocysts) and a tiny pelagic larval stage (planula larva) in its complex life cycle. These life forms are only a few millimeters or even less than one millimeter in size. The polyp is considered to be the dominant stage in life history because it usually persists throughout the whole year and reproduces asexually, while all other forms occur in response to specific conditions [26]. As most medusa populations outside China are found to be unisexual, sexual reproduction is usually absent in invasive populations. Instead, founder effects may play a role during the colonizing of new habitats by resting stages or the polyp stage which are able to cope with a large variety of environmental conditions [27,28].

Therefore, the establishment of permanent populations depends on successful colonization by long-lived polyps, which reproduce asexually and compete with resident species living in similar trophic niches. Once species arrive in a new habitat in the dispersal stage, there is no need to find a sexual partner during colonization. If polyp populations are successfully established, one or even more stimuli are required to trigger medusa budding, which is often a rare event [3]. Tiny medusae arising from asexually formed buds are then released into the water body and grow into adult jellyfish that can reproduce sexually. High densities and synchronized spawning of both sexes are needed to increase the contact rates of eggs and sperm released to the water to allow for fertilization and planula formation. There is probably a strong selection pressure on bloom formation to complete a sexual reproduction cycle. Such blooms will have a large demand for necessary resources (zooplankton) with potential top-down consequences on the local pelagic food web. During this time, resident predators, such as fishes or carnivorous zooplankton, compete for the same resources as jellyfish medusae.

Resident species that are similar to polyps of *Craspedacusta* are polyps of the genus *Hydra* (subclass Hydroidolina, family Hydridae), which are common members of benthic freshwater communities [5,29,30]. The genus *Hydra* includes 12–15 species [30,31,32] with some of them having a cosmopolitan distribution [5]. *Hydra* polyps range in height from 2 to 15 mm, have five to seven long tentacles, and attach to the substrate via an adhesive disk [33]. In comparison, *Craspedacusta* polyps are smaller (1–2 mm) and, due to lacking tentacles, show a limited capture range in comparison to *Hydra* polyps. Moreover, in contrast to *Craspedacusta*, the life cycle of *Hydra* lacks a medusa stage. Both *Craspedacusta* and *Hydra* polyps have a very similar carnivore-feeding strategy. They are passive predators waiting for potential prey touching cnidocytes on tentacles (*Hydra*) or the head of the polyp (*Craspedacusta*; [34]). Both polyps feed on crustacean zooplankton species, rotifers, oligochaete worms, nematodes, chironomids, and other insect larvae [35,36,37,38,39,40].

Similar predation strategies and food resources of *Hydra* and *Craspedacusta* polyps suggest very similar properties of their ecological niches [30] and high competitiveness. Because two functionally similar species occupying the same niche are not expected to occur at the same place at the same time (competitive exclusion principle; [41]), the question arises as to how strong trophic niches of native and invasive polyps overlap. So far, some studies show the co-occurrence of polyps from the two genera, even on the same substrate such as dreissenid mussels [30,42,43,44], which points to some niche partitioning at small scales. Unlike the permanent benthic polyp life stage, the appearance of the pelagic medusa stage is only a seasonal event. Hence, competition with other pelagic predators has mainly consequences for local food-web dynamics but not for the establishment of the species itself.

Jellyfish are primarily zooplankton predators, but they can also feed on fish [45]. They can therefore severely affect fish stocks through competition for food and predation on the next generation [46,47,48]. Traditionally, jellyfish are seen as “dead-ends”, because their high water and gelatinous mass content indicate that their food quality is low, and they have few predators compared with other zooplankton groups [49,50]. New methods such as stable isotope analyses or DNA analysis of fecal and gut samples, however, indicate that much more taxa routinely consume jellyfish and that the contribution of jellyfish to the energy budgets of predators might be higher than assumed [51].

The introduction of freshwater jellyfish created a new functional guild in invaded freshwater plankton communities outside its native range, as medusae were not represented in these lake systems before. Therefore, jellyfish are usually not included in limnic food chain concepts. Traditionally, zooplankton predators such as planktivorous fish, insect larvae, or carnivorous cladocerans are all edible prey for higher trophic levels [52]. Consequently, a very efficient food-web flow from phytoplankton to zooplankton to fish is often observed in freshwater ecosystems [53]. Similar to marine jellyfish, freshwater jellyfish are predators of a variety of prey types, such as crustacean zooplanktons, but also of insect larvae, fish eggs, and young fish [2,54,55,56,57,58]. These prey spectra suggest that jellyfish occupy a similar trophic position to planktivorous fish or native carnivorous zooplankton; however, few data are available to characterize their trophic position in situ. Moreover, even less is known about the trophic position of their benthic polyps, the stage in the jellyfish’s life cycle that occurs throughout the year [34] and whose welfare determines subsequent short-lived medusa blooms that affect the pelagic food web.

Within this study, we contribute to the understanding of the interactions that the invasive species *C. sowerbii* has with native competitors. We look at trophic food web positions in both polyp and medusa stages and compared them with those of putative competing resident predator species, using stable isotope analyses of biomass carbon and nitrogen. To cover the pelagic and benthic parts of the *Craspedacusta* life cycle, data from two studies from different regions were combined here.

The initial focus was on the bloom-forming medusa stage. A multi-year study combined with enclosure experiments revealed that *Craspedacusta* medusae are among the most important predators in the planktonic food web of Lake Alsdorf, a well-studied shallow, eutrophic lake in North Rhine–Westphalia, near Aachen [57]. With this information in mind, we hypothesize that the pelagic medusae of *Craspedacusta* occupy a similar trophic niche position to the invertebrate predator *Chaoborus* as well as zooplanktivorous fish (*Rutilus rutilus*) due to their preferred zooplanktonic diet.

Regarding the benthic stage, a wide range of prey has been reported for *Craspedacusta* polyps, including small plankton, insect larvae, nematodes, and small worms (see above), but concrete information on the extent to which there is dietary overlap with potential competitors is lacking. Here, we wanted to know whether *Craspedacusta* polyps differ in trophic niche dimensions when compared with a functionally similar and widespread native species such as *Hydra*. We used polyps of both species and potential prey from four lakes at different seasons in southern Germany and compared trophic niches and food-web position of polyps with those of other benthic predators where available. We hypothesize that *Hydra* and *Craspedacusta* polyps differ to some degree in their trophic niches, as both species can coexist in very close proximity.

## 2. Materials and Methods

To investigate the position of the pelagic and benthic life stages of *Craspedacusta* in their respective food webs, we used the available stable isotope data from separate samplings for the polyp and medusa stages. This is because medusa blooms are sporadic, and it is nearly impossible to simultaneously obtain a sufficient number of polyp samples for isotopic analyses in different lakes. In addition, the collection of polyp samples is time-consuming and requires the expertise of specialists.

### 2.1. Sampling and Sample Preparation for Pelagic Food-Web Analysis

The medusae of *Craspedacusta*, as well as samples of their potential competitors and prey, were collected in 2002 and 2003 on two dates, each in Lake Alsdorf, a small eutrophic lake with an area of 2.1 ha and a maximum depth of 4.1 m (Table A1), with nets of 2 mm mesh size. Between three and ten medusae with an umbrella diameter between 1.8 and 2.2 cm were collected per sample. The jellyfish density in 2002 and 2003 was relatively low compared with earlier years with sometimes enormously high medusa densities as well as medusa-free years [57]. Thus, 40–50 medusae per year could only be caught with intensive plankton net fishing by boat.

The larvae and pupae of the phantom midge *Chaoborus flavicans* (Diptera) were caught with plankton nets at midnight each year, as they remain in the anoxic hypolimnion near the sediment surface during the day. Head capsule length ranged from 1000 to 1250 µm, indicating the fourth larval stage (L4) of *C. flavicans* [59]. In 2002, only L4 larvae were collected (three replicates), whereas in 2003, two samples each with L4 larvae and one sample with pupae (two replicates each) were evaluated.

For the enrichment of the cladoceran species *Bosmina longirostris*, samples were pumped from a water depth of 2–2.5 m, repeatedly passed through a 160 µm sieve in the laboratory, and bosminids floating on the water surface were decanted and therefore enriched. The cladoceran species *Diaphanosoma brachyurum*, adult calanoid copepods of the species *Eudiaptomus gracilis*, and adult stages of cyclopoid copepods were enriched from plankton net samples in the laboratory. In both years, 20–25 chironomid larvae were sampled from *Cladophora*-dominated periphyton at approximately 15 cm water depth. All zooplankton samples, including jellyfish, and chironomids were rinsed three times with Millipore water before freezing.

Roach (*Rutilus rutilus*) species with a mean total length of 8 cm were caught in 2002, whereas young-of-the-year (0+) species were caught in 2003. The total length of fish was measured to the nearest 1 mm, dorsal white muscle tissue was used for isotopic analysis, and stomach contents were fixed in 90% ethanol for microscopic stomach analysis. Microscopic stomach analyses of 0+ roach species in 2003 showed similar proportions of chironomid larvae, *Chydoridae*, *Bosmina longirostris*, and copepods, which is consistent with the preference for small open-water cladocerans and chironomids from the littoral described by Densen [60]. Small roach fish of 8 cm in length, in contrast, are mostly zooplanktivorous in Lake Alsdorf when prey is suitable, preferring small cladocerans [57,61]. At the time of analysis in 2002, their stomach contents consisted almost exclusively of bosminids. Omnivorous roach fish switch from zooplanktivory to feeding on benthic resources such as detritus and macrophytes with increasing fish size [62,63]. This was also the case in Lake Alsdorf for roach species > 10 cm in 1996 and 1997 [57,61] as well as in July 2003, and therefore larger roach species were excluded from the isotope analysis, as this study focused mainly on the pelagic food chain.

Samples of zooplankton, chironomids, jellyfish, and fish were frozen on the day of sampling for later freeze-drying. Samples of pooled individuals were used for the zooplankton, chironomids, and jellyfish, whereas the fish analyses represent one individual each. After homogenization, 0.3 and 1.0 mg dry weight per sample were weighed into tin cups (cylindrical, 4 × 11 mm, Elementar Analysensysteme GmbH, Langenselbold, Germany).

Phytoplankton samples were pumped from the epilimnion and filtered through a 41 µm sieve to remove zooplankton and larger particles. Approximately 0.3 L of these plankton samples were filtered through Whatman GF/C glass fiber filters (effective pore size 0.7 µm, pre-combusted at 500 °C) and dried at 60 °C for 24 h. Parts of the filters were placed into tin cups, resulting in a weight of about 0.4–0.7 mg dry weight per sample.

During these sampling periods, the water body was thermally stable stratified, with anoxic conditions below 2.5 m (2002) and 2.0 m (2003) in both years, as already described for Lake Alsdorf in Strauss and Ratte [64] and Strauss [61] for earlier years. Epilimnetic water temperatures ranged from 21.9 to 25.4 °C, and pH values ranged from 8.0 to 8.8. In both of the years studied, the summer phytoplankton community was dominated by the N2-fixing cyanobacterial species *Aphanizomenon flos-aquae*.

### 2.2. Sampling and Sample Preparation for Benthic Food-Web Analysis

Samples of polyps from *Hydra* and *Craspedacusta* and invertebrate organisms found near the polyps were collected from four different lakes in southern Germany: Lake Hartsee (86 ha; max depth: 39.1 m), Lake Haselfurther Weiher (7.1 ha; 5.2 m), Lake Langwieder See (18.4 ha; 8.7 m), and Lake Weicheringer See (18 ha; 5 m). Each lake was sampled once between 2015 and 2016, and for Lake Langwieder See, two additional samplings were conducted to determine the seasonal changes in lakes, resulting in a total of six samples. Study sites, lake characteristics, sampling dates, and the sampled invertebrate taxa are shown in Table A1. For stable isotope analyses, whole organisms were transferred alive to tin cups (cylindrical, 5 × 9 mm, HEKAtech GmbH, Wegberg, Germany) immediately after returning from sampling. A dry weight of about 0.2 mg was needed for analyses, and a corresponding number of individuals was pooled (20 two-headed polyps from *Craspedacusta* and about 5 one-headed polyps from *Hydra*). For each taxon, at least three replicates were prepared for each sampling site and day and dried at 65 °C until weight remained constant. Additional information on the main habitat type and trophic group affiliation for the investigated pelagic and benthic taxa is shown in Table 1.

### 2.3. Stable Isotope Analysis

Stable isotope analyses are convenient tools for comparative studies of trophic niche widths and trophic positions [65,66,67,68], defining “isotopic niches” [69]. Ratios of nitrogen isotopes (^15^N:^14^N) serve to index relative trophic levels because ^15^N tends to become enriched upon increased trophic levels. On the other hand, ratios among carbon isotopes (^13^C:^12^C) are used to identify the sources of basal carbon supplies in freshwater systems [70,71]. Pelagic carbon sources in lakes are usually characterized by relatively low ^13^C:^12^C ratios compared with littoral carbon sources [72]. We used this technique to describe the niche and trophic position of *Craspedacusta* in the polyp and medusa stages.

The isotopic compositions of the dried Lake Alsdorf samples taken from the pelagic food web were determined at Leibniz Institute for Baltic Sea Research, Warnemünde, Germany, using flash combustion in a CE Instruments Flash EA 1112 Series elemental analyzer (Thermo Fisher) at 1020 °C coupled to a Thermo Finnigan MAT Deltaplus isotope ratio mass spectrometer via a Thermo Finnigan ConFlo III Interface. The isotopic values are reported relative to atmospheric N2 (δ^15^N) and Vienna PeeDee Belemnite (VPDB; δ^13^C). The reference materials used for stable isotope analysis were IAEA-N1, IAEA-N2, NBS 22, and IAEA-CH-6. The analytical precision for both stable isotope ratios was <0.2‰. The dried samples taken from the benthic food web were measured at GEOMAR (Helmholtz-Zentrum für Ozeanforschung Kiel, Kiel, Germany), following the protocols described in Hansen and Sommer [66]. The values of the stable carbon and nitrogen isotopes are presented as δ-values (‰) relative to international reference standards for carbon and nitrogen according to the equation: δ (‰) = 1000 × [(R sample/R standard) − 1].

### 2.4. Statistical Analysis

For all the analyzed taxa of the main planktonic components of Lake Alsdorf, mean δ^13^C and δ^15^N signatures (±standard errors) are reported for 2002 and 2003, respectively. For better comparability of the Lake Alsdorf results of both investigated years, the δ^13^C and δ^15^N values of the animal organisms of both years were normalized to the mean values of the isotope ratios of the phytoplankton of the respective year by subtracting the phytoplankton values from the measured values of the animal organisms (Z (taxa, isotope, year) = X (taxa, isotope, year) − X (phytoplankton, isotope, year)). To analyze the isotopic niche overlap, we used Student’s two-sample *t*-test to compare the mean δ^13^C and δ^15^N values, or the Mann–Whitney U test, as appropriate (R Development Core Team 2013). In addition, the 95% confidence intervals of the bivariate means of δ^13^C and δ^15^N were determined and compared using analyses of Stable Isotope Bayesian Ellipses in R (R Development Core Team 2013; SIBER package, Version 2.1.3, [73]) for *Craspedacusta* (medusae), *Chaoborus*, and *Rutilus* in the pelagic web, and polyps of *Hydra* and *Craspedacusta* in the benthic web. No overlap of ellipses indicates significant differences in the isotopic niches of the analyzed organisms (Table 1).

## 3. Results

### 3.1. Isotopic Niche Differentiation of Craspedacusta Medusae, Chaoborus Larvae, and Fish Larvae

**Figure 1:**
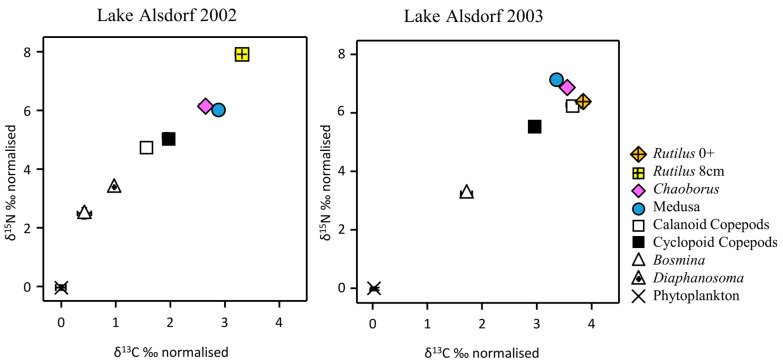
Stable isotope δ^13^C ‰ and δ^15^N ‰ signatures of main plankton food-web components in the planktonic−medusa dataset from Lake Alsdorf (mean ± SE) in 2002 and 2003. For among-year comparisons, normalized values (by phytoplankton) are shown. For details, see Table A2.

We observed clear differences in δ^13^C and δ^15^N signatures of different planktonic food-web components in Lake Alsdorf (Figure 1; Table 2). In both years, a distinction could be made in the δ^13^C and δ^15^N signatures between the basal resource in the food web (phytoplanktons), the herbivorous zooplankton (*Bosmina* and *Diaphanosoma*), and the zooplanktivorous predators (*Craspedacusta*, *Chaoborous*, and *Rutilus*) (Figure 1).

Predatory taxa were ^15^N-enriched relative to herbivorous consumers. The enrichment of ^15^N between trophic levels from phytoplankton to phytoplanktivorous *Bosmina* was 2.72‰ (2002) and 3.28‰ (2003), respectively. *Craspedacusta* and *Chaoborus* were ^15^N-enriched by 3.61 to 3.95‰ compared with *Bosmina*. Compared with *Bosmina, Rutilus* 0+ was enriched by 3.12‰, and *Rutilus* 8 cm by 5.43‰. On average, the ^15^N-enrichment was around 3.7‰ per trophic level. The δ^15^N isotopic signatures for calanoid copepods (*Eudiaptomus gracilis*) were similar to or even higher than the signatures of cyclopoid copepods. While the δ^15^N values were quite stable among taxa between the two years, the 2002 and 2003 data of δ^13^C were markedly different for some taxa: *Bosmina*, as well as cyclopoid and calanoid copepods (Figure 1 and Table A2). However, this was not the case for *Chaoborus* or *Craspedacusta*, both of which had very similar δ^13^C signatures when comparing the two years. The ^13^C enrichment between trophic components was between 0.9‰ and 2.43‰ (mean 1.63‰).

**Figure 2:**
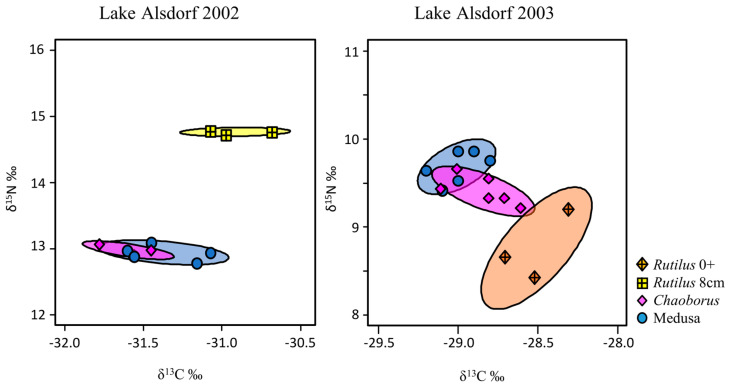
SIBER analysis of stable isotope δ^13^C ‰ and δ^15^N ‰ signatures of taxa from the planktonic−medusa dataset of Lake Alsdorf. Ellipses depict 95% confidence intervals. No overlap of ellipses indicates significant differences in the isotopic niches between taxa.

Generally, the evaluation based on SIBER (Figure 2) and the *t*-test results (Table 2) mutually confirmed each other. The signatures of the stable isotopes of *Craspedacusta* and *Chaoborus* showed no significant differences, with completely overlapping niche ellipses in 2002 (Figure 2). In 2002, the *Rutilus* signature was significantly different from the *Craspedacusta*–*Chaoborus* cluster. In particular, the δ^15^N-signature was significantly higher (Figure 1 and Figure 2). In 2003, the *Rutilus* 0+ signature was relatively similar to the invertebrate competitors (Figure 2).

### 3.2. Isotopic Niche Differentiation of Craspedacusta and Hydra Polyps

**Figure 3:**
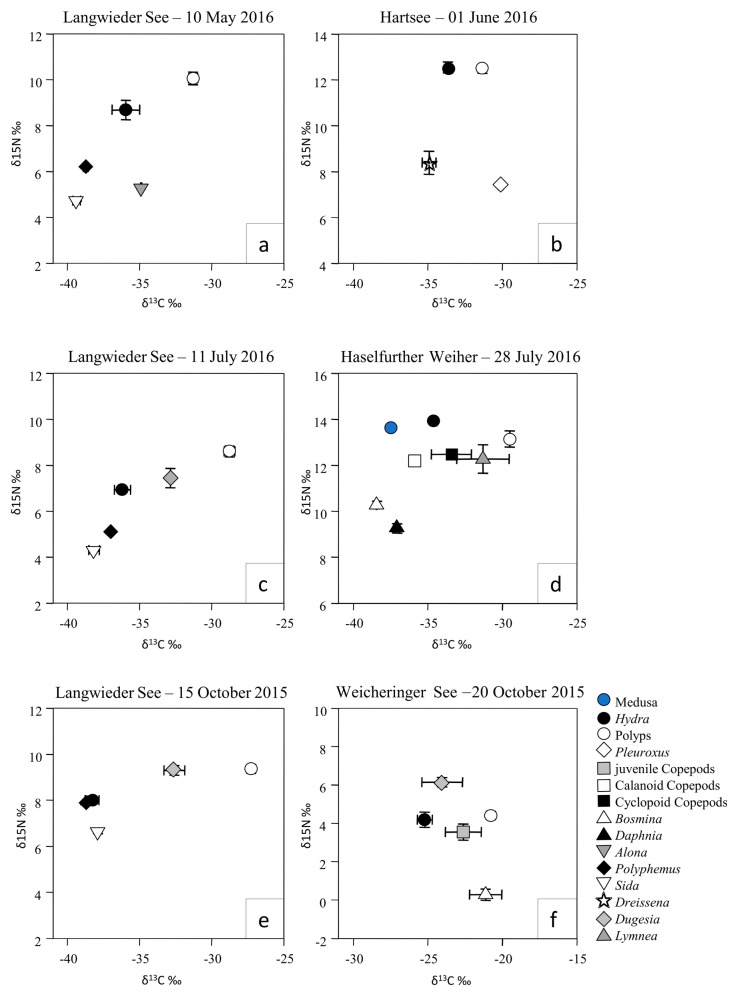
Stable isotope δ^13^C ‰ and δ^15^N ‰ signatures of taxa from the benthic−polyp dataset (mean ± SE) from Lake Langwieder See (**a**,**c**,**e**), Lake Hartsee (**b**), Lake Haselfurther Weiher (**d**), and Lake Weicheringer See (**f**). Medusa: *Craspedacusta* medusae; polyps: *Craspedacusta* polyps; *Hydra: Hydra* polyps.

In all four lakes studied for benthic food-web analysis, and at all sampling dates, δ^13^C signatures differed between polyps of *Craspedacusta* and *Hydra* (Figure 3, Table A3). Notably, the mean δ^13^C values of polyps of *Craspedacusta* were similar to the mean δ^13^C values of the littoral herbivores *Pleuroxus truncatus* and *Lymnea stagnalis* (Figure 3b,d). In comparison, the δ^13^C values of *Hydra* were in the same range as those for pelagic cladocerans *Polyphemus pediculus, Sida crystallina*, *Alona* sp., *Daphnia* sp., and of the zebra mussel *Dreissena polymorpha* (Figure 3b). Additionally, the δ^13^C values of the pelagic carnivore free-swimming medusae of *Craspedacusta* were more similar to the δ^13^C values of *Hydra* than to the values of the polyps of *Craspedacusta*.

The δ^13^C values of the benthic carnivore *Dugesia* sp. (Plathelmintha) and of copepods (pelagic omnivore) were within or in between the range of both polyp genera (Figure 3). The δ^15^N values of the two polyp genera were only significantly different in two of the sampling dates (Table A3). At four of the sampling dates, no significant differences were observed in the δ^15^N signatures among polyps of *Craspedacusta* and *Hydra*.

**Figure 4:**
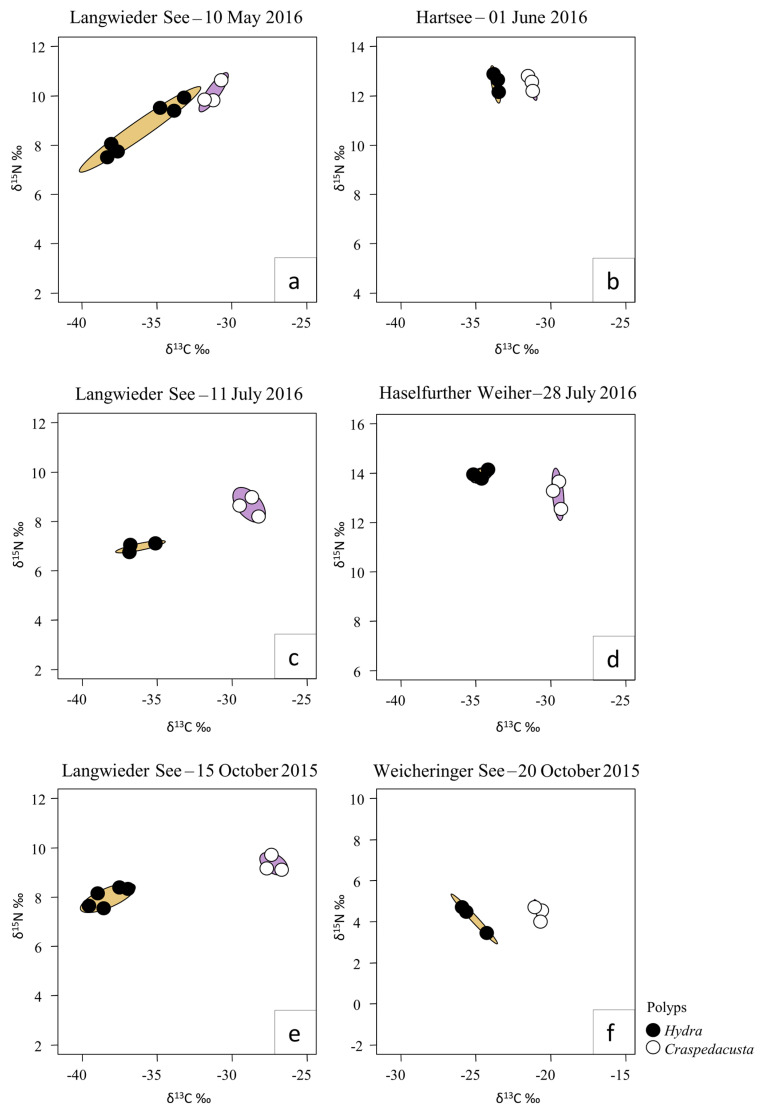
SIBER analysis of stable isotope δ^13^C ‰ and δ^15^N ‰ signatures from the benthic−polyp dataset. Ellipses depict 95% confidence intervals. No overlap of ellipses indicates significant differences in the isotopic niches between *Craspedacusta* polyps and *Hydra* polyps from Lake Langwieder See (**a**,**c**,**e**), Lake Hartsee (**b**), Lake Haselfurther Weiher (**d**), and Lake Weicheringer See (**f**).

Similar results were obtained from SIBER analyses using data from all six sampling dates (Figure 4). Ellipses displaying 95% confidence intervals based on the bivariate means of δ^13^C and δ^15^N signatures did not overlap for polyps of *Craspedacusta* and *Hydra*. Interestingly, there was an interaction between season and trophic niche dimensions in Lake Langwieder See, where the trophic niche ellipses almost overlapped in May (Figure 4a) but increasingly diverged from July (Figure 4c) to October (Figure 4e).

## 4. Discussion

*Craspedacusta* is a highly invasive freshwater jellyfish and probably one of the most successful freshwater invaders globally [18]. We investigated the trophic niche characteristics of the medusa and polyp stage of *Craspedacusta* as well as functionally similar native species, the pelagic zooplanktivorous competitors *Chaoborus flavicans* and roach (*Rutilus rutilus*), and benthic *Hydra* polyps.

### 4.1. Trophic Position of Craspedacusta Medusae

The very similar isotopic signatures of *Craspedacusta* medusae and *Chaoborus* indicate a strong niche overlap due to the use of similar food resources and the formation of a common trophic cluster. This suggests that, at least in Lake Alsdorf, they form a trophic cluster and use very similar food sources. In contrast, the signatures of *Rutilus* showed a somewhat different, more complex picture. While the 2003 0+ cohort, which also fed on benthic organisms in addition to zooplankton, as revealed through stomach analysis, showed a more similar isotopic signature to the *Craspedacusta*–*Chaoborus* cluster, the juvenile *Rutilus* larger than 8 cm, from 2002, that were zooplanktivorous at the time of sampling showed a distinctly different isotopic signature, with higher δ^15^N values.

In particular, larger consumers such as fish may deviate from isotopic equilibrium with their current diet due to the isotopic “memory” of prey assimilated at earlier times, since isotopic ratios, in addition to diet and fractionation, also depend on consumer growth patterns, and a change in isotopic composition is dominated by the addition of new tissue [77,78]. However, in addition to the intake of protein-rich foods during the growth phase, starvation can also increase the δ^15^N signature [79]. The δ^15^N signature of 0+ roach species, on the other hand, may have been lowered by the uptake of benthic resources such as chironomid larvae, with relatively low δ^15^N content, as was also found in Lake Alsdorf (Table A2). Although the above reasons make isotopic comparisons of juvenile roach fish with the other invertebrate predators in Lake Alsdorf difficult, the results show that juvenile and mostly zooplanktivorous roach are not appreciably higher in the food chain than *Craspedacusta* and *Chaoborus*. This is also supported by the findings of Harrod and Grey [80], according to which *Chaoborus* had only slightly lower mean δ^15^N values than roach fish at overlapping signature ranges.

In our present study, comparable to the Lake Alsdorf data, *Craspedacusta* medusae from Lake Haselfurther Weiher were ranked in the top food-web position based on isotopic signatures (Figure 3d), showing consistency among the different locations. The results of the stable isotope studies confirm the conclusions from previous lake enclosure experiments [57] indicating that *Craspedacusta* medusae are among the top predators in the planktonic food web of Lake Alsdorf. This finding is also supported by studies of another freshwater jellyfish at Lake Tanganyika. The trophic position, as revealed by the stable isotopes of the freshwater medusa *Limnocnida tanganyicae*, is comparable and is suggested to have a strong top-down influence on zooplankton, particularly during blooms of medusae [81].

*Craspedacusta* medusae show clear food preferences for copepods and their nauplius larvae, although small cladocerans are also positively selected; small *Chaoborus* larvae (<3 mm) may occasionally be part of their diet, whereas small rotifers tend to be negatively selected [56,57,82]. Since the food prey range is limited to a size of about 5 mm [56], the fourth instar larvae (about 10 mm) of *Chaoborus* are not appropriate prey anymore. *Chaoborus flavicans* shows a very similar food preference, with a strong positive selection of copepods (especially those in copepodite stages) and nauplii, as well as small- and medium-sized cladocerans [83], while small rotifers may also be important part of the diet for fourth instar larvae [84]. In contrast, our own studies from Lake Alsdorf [57] revealed a strong positive selection for cladocerans in young roach species, although copepods are also occasionally ingested, with much less preference. From these food preferences, it can be concluded that fish, medusae, and *Chaoborus* strongly compete for cladocerans, while mainly medusae and *Chaoborus* compete for copepods.

Temporal separation at a perennial level may contribute to *Chaoborus* co-occurring with *Craspedacusta*. The appearance of *Craspedacusta* medusae in lakes is not only very irregular but also very sporadic. There are several observations in which medusae showed very high abundances for only one or two consecutive years and then did not reappear for years [27,38,57,85,86,87] or only appeared in a very small number in some years, as in the years studied here. If medusae have a negative impact on *Chaoborus* during a mass occurrence event through food competition and predation, then only sporadic occurrence of medusae would allow *Chaoborus* populations to recover.

### 4.2. Isotopic Niche Differentiation of Craspedacusta Polyps

The ecological success of the polyp stage of *Craspedacusta* is decisive in the establishment of the invasive species because medusae represent reproductive dead-ends outside their native range (see below). *Craspedacusta* polyps have potential native cnidarian competitors such as *Hydra* polyps, with possible implications for interspecific competition and long-term competitive exclusion. Indeed, small-scale co-occurrence of polyps from both genera has been frequently reported, and both share similar dietary niches [30,42,43,44]. Hence, one would assume strong interspecific competition for food.

*Hydra* and *Craspedacusta* showed a similar trophic position: The differences between the δ^15^N values of the two polyp types were on average 0.92‰. These small differences support the assumption that the two polyp genera are similar in their trophic level and clearly predatory. The δ^15^N values of the two polyp genera were around 1 to 1.5 trophic positions higher than the ones for herbivore benthic filter-feeder *Dreissena polymorpha* or herbivore pelagic filter-feeder *Daphnia longispina*, assuming a δ^15^N difference of around 2‰ per trophic level [74]. However, the mean δ^13^C values of the two different polyps were significantly different, and the isotopic niche widths never overlapped (Table A3; Figure 4). This clearly shows that the two polyp types have different dietary carbon sources and are not competing for the same food. *Craspedacusta* polyps had consistently heavier carbon signals than the *Hydra* polyps. The δ^13^C values of *Hydra* were in general closer to δ^13^C values of the free-swimming medusae of *Craspedacusta* and the filter-feeding cladocerans *Sida crystallina, Alona* sp. and *Daphnia longispina.* The carbon sources of these taxa are predominantly pelagic [34,74], reflected by their relatively lighter δ^13^C values compared with mainly benthic grazers such as the cladoceran species *Pleuroxus truncatus* [75] or gastropods such as *Lymnea stagnalis* [88]. As the δ^13^C signature of *Craspedacusta* polyps was similar to the values of these benthic grazers, their carbon source was also mostly benthic. Such strong niche separation between *Hydra* and *Craspedacusta* polyps was observed in all the investigated lakes and at all seasonal sampling dates. This general pattern can be explained by the morphological differences in the two polyp genera. Due to the highly flexible and elongated body with long tentacles, *Hydra* polyps are able to catch pelagic prey, whereas polyps of *Craspedacusta* are largely limited to benthic prey organisms due to their smaller size and lack of tentacles. Such resource partitioning based on morphology as observed in our study is known to allow for the coexistence of competing species [24]. The trophic niche of *Hydra* seems to be larger in spring (May) than in July or October, as seen in the seasonal sampling approach at Lake Langwieder See (Figure 4). This may result from higher abundances of different crustacean species in spring than in summer or autumn, when this prey type is reduced due to high predation pressure by fish [89,90]. The seasonal sampling at Lake Langwieder See showed that the size of the dietary niche of both polyps varied seasonally, thereby resulting in different strength of dietary niche separation.

However, despite some obvious flexibility in niche separation, the two polyp types occupy separate dietary niches, independently of season and lake types, which should enable their coexistence. Our study indicates that the establishment of *Craspedacusta* should not be affected by competition for food with functionally similar native Cnidaria types represented by *Hydra* polyps.

### 4.3. Trophic Niches in the Metagenetic Life Cycle of Craspedacusta

Our study combined data on the trophic role of polyps and medusae of the freshwater jellyfish *Craspedacusta* within benthic and pelagic food webs of lakes. In both systems, *Craspedacusta* was clearly a top predatory species.

Medusa formation, a prerequisite for sexual reproduction in *Craspedacusta,* is a sporadic event [3] connecting benthic and pelagic habitats in lakes. It is the time window in which, under favourable conditions, medusa buds are formed in the polyps and the juvenile medusae are released into the pelagial. Within the pelagic food web, medusae compete with resident predators for similar resources such as *Chaoborus* or small fishes, as reflected in their strongly overlapping trophic niches. Notably, the interplay of community settings, ecology, and physical factors altogether may determine successful medusa formation, which is not fully predictable yet. However, with the production of medusae and the development of jellyfish blooms, major impacts on the food-web structure can be seen. The sporadic character of such events may prevent the competitive exclusion of other carnivorous zooplankton or have strong and long-lasting effects on fish populations.

In contrast to pelagic medusae, polyps showed a clear difference in their trophic niches when compared with their native cnidarian competitors. It can be assumed that the benthic part of the *Craspedacusta* life cycle promotes the establishment of local populations since even a single immigrant polyp haplotype can form a big pool of polyp colonies over time through steady asexual reproduction. Then, polyp competitiveness is the key to the long-term establishment of populations, with subsequent benthic–pelagic habitat coupling via the production of medusae. We found that the isotopic signatures of benthic *Craspedacusta* polyps significantly differ from resident *Hydra* polyps and from other benthic predators such as *Dugesia*, suggesting low interspecific competitive pressure for this immigrant predator.

## 5. Conclusions

Overall, the presented data on trophic niches of the different life stages of *Craspedacusta* help to explain its large invasive success. Biotic interactions with competitors and predators can shape the fundamental niche of a species—determined by its need for resources and the tolerance of environmental conditions—into a much smaller “realized” niche. In the case of *Craspedacusta,* the realized niche might be not too different from the fundamental niche in the polyp stage, as the competitive exclusion of the species by native competitors probably might not occur yet.

## Figures and Tables

**Table 1:** Taxa sampled for stable isotope analyses. P: planktonic−medusa dataset from Lake Alsdorf; B: benthic−polyp dataset from Lake Hartsee, Lake Haselfurther Weiher, Lake Langwieder See, and Lake Weicheringer See. The main habitat and trophic group affiliations of invertebrates are presented according to [34,74,75,76].

Dataset	Symbol	Taxon	Main Habitat and Trophic Group Affiliations
B	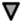	*Alona* sp.	littoral filter-feeder, herbivore
P, B	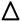	*Bosmina* sp.	pelagic filter-feeder, herbivore
P, B	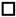	Calanoid Copepods (adults)	pelagic, herbivore/omnivore
P	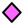	*Chaoborus flavicans*	pelagic, carnivore
B	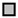	Copepods (juveniles)	pelagic, omnivore
P, B	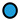	*Craspedacusta* Medusa	pelagic, carnivore
B	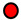	*Craspedacusta* Polyp	benthic, carnivore
P, B	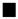	Cyclopoid Copepods (adults)	pelagic, carnivore/omnivore
B	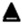	*Daphnia longispina*	pelagic, filter-feeder, herbivore
P	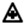	*Diaphanosoma brachyurum*	littoral/pelagic, filter-feeder, herbivore
B	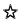	*Dreissena polymorpha*	benthic, filter-feeder
B	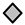	*Dugesia* sp.	benthic, carnivore
B	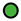	*Hydra vulgaris*	benthic, carnivore
B	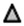	*Lymnea stagnalis*	littoral/pelagic, filter-feeder, omnivore
B	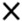	Phytoplankton (<41 µm)	pelagic, primary producers
B	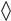	*Pleuroxus truncatus*	littoral, herbivore
B	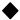	*Polyphemus*	pelagic, carnivor
P	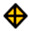	*Rutilus rutilus* 0+ (total length 3.6–4.9 cm)	pelagic, zooplanktivore/omnivore
P	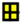	*Rutilus rutilus* 8 cm (total length 7.5–8.2 cm)	pelagic, zooplanktivore/omnivore
B	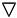	*Sida crystallina*	littoral, filter-feeder. herbivore

**Table 2:** Niche differentiation of *Craspedacusta, Chaoborus*, and *Rutilus* (0+ and 8 cm) in the pelagic food web. Shown are *p*-values from pairwise comparisons (*t*-test) of δ^13^C ‰ and δ^15^N ‰ signatures for 2002 (above the diagonal) and 2003 (below the diagonal, in italics). Significant *p*-values in bold.

	δ^13^C ‰			δ^15^N ‰		
	*Craspedacusta*	*Chaoborus*	*Rutilus* 8 cm	*Craspedacusta*	*Chaoborus*	*Rutilus* 8 cm
*Craspedacusta*	---	>0.05	***<0.05***	---	>0.05	***<0.001***
*Chaoborus*	*>0.05*	---	***0.01***	***<0.05***	---	***<0.001***
*Rutilus* 0*+*	***<0.05***	***<0.05***	---	***<0.01***	***<0.01***	---

## Data Availability

The data presented in this study are available on request from the corresponding author.

## Appendix A

**Table A1:** Taxa sampled for stable isotope analyses in the planktonic−medusa (Lake Alsdorf) and benthic−polyp datasets from four lakes. Superscript numbers refer to different sampling dates within lakes. a: Epilimnetic total phosphorus during July–August 1995–1997 (means ± SD, data from [61]).

	Lake Alsdorf	Lake Hartsee	Lake Haselfurther Weiher	Lake Langwieder See	Lake Weicheringer See
Latitude	50.8626	47.9268	48.4820	48.1969	48.7036
Longitude	6.1531	12.3671	12.0130	11.4166	11.3296
Max. depth (m)	4.1	39.1	5.2	8.7	5.0
Area (m^2^)	31,000	860,000	70,500	184,000	180,000
TP (µg L^−1^)	164 ± 36 ^a^	13.58	8.48	8.20	11.88
Lake type	pond	natural lake	quarry pond	quarry pond (since 1930)	quarry pond (since 1965)
Sampling dates	^(1)^ 30 July 2002				
	^(2)^ 02 August 2002				
	^(3)^ 05 August 2002	1 June 2016	28 July 2016	^(1)^ 15 October 2015	20 October 2015
	^(4)^ 15 July 2003			^(2)^ 10 May 2016	
	^(5)^ 18 July 2003			^(3)^ 11 July 2016	
	^(6)^ 21 July 2003				
	^(7)^ 26 July 2003				
Sampled invertebrate taxa and fish	Phytoplankton ^(3,6)^	*Craspedacusta* Polyps	*Craspedacusta* Polyps	*Craspedacusta* Polyps ^(1,2,3)^	*Craspedacusta* Polyps
	*Bosmina longirostris* ^(3,7)^	*Hydra* Polyps	*Hydra* Polyps	*Hydra* Polyps ^(1,2,3)^	*Hydra* Polyps
	*Diaphanosoma brachyurum* ^(3)^	*Pleuroxus truncatus*	*Bosmina* sp.	*Alona* sp. ^(2)^	*Bosmina* sp.
	*Eudiaptomus gracilis* ^(3,6,7)^	*Dreissena polymorpha*	*Daphnia longispina*	*Polyphemus pediculus* ^(1,2,3)^	Calanoid and cyclopoid copepods
	Cyclopoid copepods ^(3,6,7)^		Calanoid copepods	*Sida crystallina* ^(1,2,3)^	*Dugesia* sp.
	Chironomid larvae ^(3,6)^		Cyclopoid copepods	*Dugesia* sp. ^(1,3)^	
	*Craspedacusta sowerbii* Medusae ^(1,2,4,5)^		*Lymnea stagnalis*		
	*Chaoborus flavicans* ^(3,6)^		*Craspedacusta* Medusa		
	*Rutilus rutilus* 8 cm ^(3)^				
	*Rutilus rutilus* 0+ ^(7)^				

**Table A2:** Isotopic data of main planktonic components (except rotifers, but with Chironomids) and fish from Lake Alsdorf. Shown are δ^13^C and δ^15^N signatures (mean ± SE), N: sample size.

Taxon	Year	N	δ^13^C ‰	δ^15^N ‰
*Bosmina*	2002	4	−33.80 ± 0.13	9.33 ± 0.18
Calanoida (adult)	2002	2	−32.72 ± 0.03	11.65 ± 0.04
*Chaoborus*	2002	3	−31.60 ± 0.10	12.98 ± 0.04
*Chironomid* larvae	2002	3	−33.82 ± 0.11	6.79 ± 0.05
*Craspedacusta* Medusae	2002	5	−31.37 ± 0.11	12.94 ± 0.04
Cyclopoida (adult)	2002	4	−32.30 ± 0.07	11.95 ± 0.11
*Diaphanosoma*	2002	2	−33.27 ± 0.04	10.26 ± 0.02
Phytoplankton	2002	5	−34.22 ± 0.10	6.83 ± 0.06
*Rutilus* 8 cm	2002	3	−30.92 ± 0.12	14.76 ± 0.02
*Bosmina*	2003	4	−30.63 ± 0.10	5.88 ± 0.03
Calanoida (adult)	2003	4	−28.73 ± 0.08	8.90 ± 0.15
*Chaoborus*	2003	6	−28.83 ± 0.08	9.58 ± 0.06
*Chironomid* larvae	2002	3	−30.33 ± 0.15	5.27 ± 0.07
*Craspedacusta* Medusae	2003	6	−29.00 ± 0.06	9.83 ± 0.07
Cyclopoida (adult)	2003	4	−29.40 ± 0.04	8.20 ± 0.15
Phytoplankton	2003	5	−32.34 ± 0.06	2.60 ± 0.05
*Rutilus* 0+	2003	3	−28.50 ± 0.12	9.00 ± 0.21

**Table A3:** Niche differentiation in the benthic food web between *Craspedacusta* and *Hydra* polyp samples from four lakes. Shown are δ^13^C and δ^15^N signatures (mean ± SE) and *p*-values from pairwise comparisons (*t*-test, N = 3). Significant *p*-values in bold.

		δ^13^C ‰			δ^15^N ‰		
Lake	Date	*Craspedacusta*	*Hydra*	*p*	*Craspedacusta*	*Hydra*	*p*
Langwieder See	10 May 2016	−31.3 ± 0.3	−36.0 ± 0.9	**0.024**	10.1 ± 0.3	8.7 ± 0.4	0.069
	11 July 2016	−28.8 ± 0.4	−36.2 ± 0.6	**<0.001**	8.6 ± 0.2	6.9 ± 0.1	**0.002**
	15 October 2015	−27.3 ± 0.3	−38.3 ± 0.5	**<0.001**	9.4 ± 0.2	8.0 ± 0.2	**0.002**
Hartsee	1 June 2016	−31.3 ± 0.1	−33.6 ± 0.1	**<0.001**	12.5 ± 0.2	12.5 ± 0.2	0.825
Haselfurther Weiher	28 July 2016	−29.5 ± 0.1	−34.6 ± 0.2	**<0.001**	13.1 ± 0.4	13.9 ± 0.1	0.100
Weicheringer See	20 October 2015	−20.8 ± 0.1	−25.2 ± 0.5	**<0.001**	4.4 ± 0.2	4.2 ± 0.4	0.636
